# Effects of CaCl_2_ on 3D Printing Quality of Low-Salt Surimi Gel

**DOI:** 10.3390/foods12112152

**Published:** 2023-05-26

**Authors:** Chaoye Wang, Mengjie Ma, Yabo Wei, Yunfeng Zhao, Yongdong Lei, Jian Zhang

**Affiliations:** 1 School of Food Science and Technology, Shihezi University, Shihezi 832003, China; 20202111024@stu.shzu.edu.cn (C.W.);; 2 Key Laboratory for Processing and Quality Safety Control of Specialty Agricultural Products of Ministry of Agriculture and Rural Affairs, Shihezi 832003, China; 3 Key Laboratory for Food Nutrition and Safety Control of Xinjiang Production and Construction Corps, Shihezi 832003, China

**Keywords:** surimi gel, 3D printing, salt substitute, gel property, product quality

## Abstract

In order to develop low-salt and healthy surimi products, we limited the amount of NaCl to 0.5 g/100 g in this work and studied the effect of CaCl_2_ (0, 0.5, 1.0, 1.5, and 2.0 g/100 g) on the 3D printing quality of low-salt surimi gel. The results of rheology and the 3D printing showed that the surimi gel with 1.5 g/100 g of CaCl_2_ added could squeeze smoothly from the nozzle and had good self-support and stability. The results of the chemical structure, chemical interaction, water distribution, and microstructure showed that adding 1.5 g/100 g of CaCl_2_ could enhance the water-holding capacity and mechanical strength (the gel strength, hardness, springiness, etc.) by forming an orderly and uniform three-dimensional network structure, which limited the mobility of the water and promoted the formation of hydrogen bonds. In this study, we successfully replaced part of the salt in surimi with CaCl_2_ and obtained a low-salt 3D product with good printing performance and sensory properties, which could provide theoretical support for the development of healthy and nutritious surimi products.

## 1. Introduction

3D printing, defined as a technology based on digital model files to construct objects by the successive addition of material layers, is an emerging technology combining digital technology and computer technology [1,2,3]. It was first reported in 1986 and was gradually applied in the food sector in the early 2000s, which is considered a sign of the beginning of the third industrial revolution [4,5]. The application of 3D printing technology to the food field can not only reduce costs and improve efficiency but also realize a scientific diet structure, provide customized nutritional formulas, and give food a unique texture so as to meet the personalized needs of consumers for food nutrition and appearance [6]. At present, extrusion printing based on the principle of fused deposition modeling (FDM) is the most widely used in the food field [7,8,9].

With the development and innovation of 3D food printing technology, it has been found that fish surimi, as an intermediate product of fish processing, is an ideal raw material suitable for extrusion-based 3D printing. It can be used to produce 3D surimi products with customized nutrients and textures to meet the needs of different people. However, its 3D-printed products must be cooked before being edible, and different post-processing methods have a great impact on product quality. At the same time, to obtain a gel performance suitable for extrusion printing, 1.0–3.0 g/100 g of NaCl is customarily added to gels [10]. Sodium chloride (NaCl) can promote the dissolution of myofibrillar proteins and the expansion of proteins’ structure, making the proteins combine with more water molecules to form a three-dimensional network structure so as to enhance the strength of the surimi gel and improve its rheological characteristics [11]. At the same time, NaCl also gives foods a unique flavor. Current studies have shown that we consume too much salt (9–12 g/day), about twice as much as the daily salt intake (≤5 g) per person recommended by the WHO [12]. The evidence has indicated that excessive sodium intake is associated with the occurrence of hypertension and cardiovascular disease (CVD) [13]. Therefore, it is necessary to reduce the amount of NaCl added to existing gel formulations to develop delicious, healthier foods. However, cutting down the amount of NaCl would weaken the gel property, and improving the health benefits of surimi gels should not be at the expense of quality [14]. Some studies have reported that the addition of some accessories, such as κ-carrageenan, CaCl_2_, transglutaminase (TGase), and so on, can improve surimi gel’s performance and make it suitable for extrusion printing [11,15,16]. Calcium ion (Ca^2+^) can promote the unfolding of proteins by weakening the intra-molecular hydrophobic interactions, exposing some functional groups, and enhancing the cross-linking of proteins through hydrophobic interactions and disulfide bonds [17,18]. With the concentration of Ca^2+^ increasing, Ca^2+^ can promote the formation of a calcium bridge and the further aggregation of protein molecules, thus enhancing the gel strength of surimi [12]. Additionally, the endogenous TGase activity of surimi can be activated by Ca^2+^ to catalyze the intra- and inter-molecular covalent cross-linking of the proteins [19,20]. TGase can effectively improve the viscoelastic and mechanical properties of surimi by inducing the formation of ε-(γ-Glu)Lys, promoting protein aggregation, and forming a gel [21]. Moreover, it can also improve the stability of printed products after heating, making it easier to retain the shape features of products.

In order to improve the quality of surimi gel and compensate for the poor characteristics of low-salt surimi gel (0.5 g/100 g), TGase and different concentrations of CaCl_2_ were added to *Esox lucius* surimi in this study. The effects of CaCl_2_ on the 3D printing quality of low-salt surimi gel were studied by determining the rheological properties of surimi gels and the printing accuracy, shrinkage, cooking loss, water-holding capacity, whiteness, gel strength, texture, chemical interactions, chemical structures, water distribution, and microstructure of 3D-printed products. The results of this study can provide a theoretical basis for the development of low-salt surimi products.

## 2. Materials and Methods

### 2.1. Materials

*Esox lucius* (1.0–1.5 kg) was purchased from a local market in Urumqi, China.

### 2.2. Preparation of Surimi and Surimi Gel

#### 2.2.1. Preparation of Surimi

After being anesthetized to death, the fish was manually descaled, gutted, decapitated, and the bones were removed. Then, the fish skin was removed with a peeling machine (SC-1000, Wanlong Machinery Co., Ltd., Foshan, China), and the fish meat was crushed into minced meat by a meat separator (XZC-220, Xuzhong Food Machinery Co., Ltd., Guangzhou, China) with a 4-mm-diameter hole plate, whereafter, the minced meat was washed 3 times with 4 °C ice water (1:3, *w*/*w*) and then squeezed to remove water by putting it into a nylon bag (200-mesh). Next, the moisture content of surimi was concentrated to below 80% by freeze-drying at −50 °C for 36 h. Finally, the surimi was mixed well, sealed in the package, and stored at −24 °C.

#### 2.2.2. Preparation of Surimi Gel

After weighing a certain amount of surimi, 0.5 g/100 g of NaCl, 0.2 g/100 g of TGase (100 U/g), and different concentrations of CaCl_2_ (0, 0.5, 1.0, 1.5, and 2.0 g/100 g, respectively) were added, the moisture content was adjusted to 82 g/100 g with ice water, and this was followed by chopping it in a blender (JYL-C012, Jiuyang Co., Ltd., Jinan, China) for 5 min in order to mix evenly. In the process of mixing, the blender was shut down for 40 s for every 20 s run in order to keep the temperature in the blender below 10 °C so as to avoid the formation of a thermal gel and ensure the quality of the slurry [22]. Moreover, it was also convenient to scrape the surimi adhered to the inner wall of the blender to the bottom to ensure uniform cutting. The formulated surimi gels were transferred into printer syringes and then stored at 4 °C for 4 h before 3D printing. The bubbles in the syringes were excluded by centrifugation.

### 2.3. Determination of Rheological Properties

The rheological properties of surimi gel were measured by a rheometer (MCR-302, Anton Paar, Graz, Austria) at 25 °C. A parallel plate (20-mm-diameter) was used with a gap of 1000 μm. The surimi gel was placed on a platform and equilibrated for 1 min before measurements [21]. For the determination of apparent viscosity, the flow sweep mode was applied, and the shear rate ranged from 0.1 to 100 s^−1^. The dynamic viscoelastic property was determined in the oscillation frequency scanning mode. The angular frequency was oscillated from 0.1 to 100 rad/s, and all measurements were performed at a 1% strain (identified within the linear viscoelastic region). The storage modulus (G′) and loss modulus (G″) were recorded.

### 2.4. 3D Printing Processing and Performance

The cartridge with the formulated surimi gel was loaded into the extrusion system of a 3D printer (FoodBot-D1, Shiyin Technology Co., Ltd., Hangzhou, China). Then, the 3D digital model (cylinder, diameter = 25 mm, height = 20 mm), designed with 123D Design (Autodesk, Inc., San Francisco, CA, USA), was sliced by Sli3r software, V1.2.9, and then converted into a 3D physical sample. The printing progress was performed at room temperature (25 °C), and the printing parameters were set as the following: 1.2-mm nozzle diameter, 1.1 mm-layer height, 100% infill density, a concentric infill pattern, and 15 mm/s speed (60% for the first layer speed).

Face-view and top-view digital photos of the 3D samples were collected immediately after the 3D printing was completed. Then, the samples were cooked in a two-step heating manner: first, the samples were heated in a 40 °C water bath for 60 min, and then they were heated for 30 min in a 90 °C water bath [23]. Subsequently, the cooked samples were cooled in ice water for 15 min, and the moisture on the surface of the samples was gently dried with absorbent paper. The face-view and the top-view images of cooked samples were collected.

### 2.5. Printing Precision and Shrinkage

The dimensions of samples before and after cooking were measured using a digital vernier caliper (DL3944, Deli Tool Co., Ltd., Ningbo, China) in three replicates to estimate the printing performance. The printing precision and shrinkage were calculated by the following formulas:

*Printing precision* (%) = [1 − (*D*_1_ − *D*_0_)/*D*_0_] × 100

*Shrinkage* (%) = [(*D*_1_ − *D*_2_)/*D*_1_] × 100

where *D*_0_ is the dimension of the 3D model, *D*_1_ is the dimension of the 3D-printed sample, and *D*_2_ is the dimension of the heated 3D-printed sample.

### 2.6. Cooking Loss and Water-Holding Capacity (WHC)

The cooking loss of 3D samples was determined according to the method of Dick et al. [24], which was calculated by weighing the mass of the samples before cooking (*m*_0_) and after cooking (*m*_1_), expressed as follows:

*Cooking loss* (%) = [(*m*_0_ − *m*_1_)/*m*_0_] × 100

The determination of the *WHC* followed the method described by Yu et al. [19], in which approximately 2 g of cooked samples were centrifuged at 10,000× *g* for 15 min at 4 °C. The *WHC* (%) was calculated by the following formula:

*WHC* (%) = (*Y*/*X)* × 100

where *X* is the mass of the sample before centrifuging, and *Y* is the mass of the sample after centrifuging.

### 2.7. Color Evaluation

The color of the cooked 3D samples was measured by utilizing a colorimeter (SC-10, 3nh Technology Co., Ltd., Shenzhen, China), and the CIE L* (lightness), a* (redness/greenness), and b* (yellowness/blueness) values were recorded, respectively [25]. Whiteness was calculated by the following equation:

*Whiteness* = 100 − [(100 − L*)^2^ + a*^2^ + b*^2^]^1/2^

### 2.8. Determination of Gel Strength and Texture Profile Analysis (TPA)

According to the methods of Zhao et al. and Zhang et al., with slight modifications, the gel strength and TPA of cooked 3D samples were both determined at room temperature (25 °C) using a texture analyzer (TA-XT plus, Stable Micro Systems, Surrey, UK) [26,27]. For the gel strength, a puncture test was performed using a spherical probe (P/0.25S) with a test speed of 1 mm/s and a strain of 75%. The breaking force (N) and breaking distance (mm) were obtained from the force–displacement curve. The gel strength (N·mm) is expressed as the product of the breaking force and the breaking distance. For TPA, a double-cycle compression test was performed utilizing a cylindrical probe (P/50) at a test speed of 1 mm/s, a strain of 50%, and a residence time of 3 s. Hardness, springiness, cohesiveness, chewiness, and resilience were recorded.

### 2.9. Low-Field Nuclear Magnetic Resonance (LF-NMR)

The transverse relaxation time (*T*_2_) of the cooked sample was measured using a LF-NMR food analyzer (PQ001-20-025V, Niumag Analytical Instrument Corporation, Suzhou, China), according to the methods of Chen et al. and Dong et al., with modifications [21,28]. Briefly, 2 g of cooked samples were wrapped with plastic wrap and put into a 25-mm NMR tube, followed by equilibration at room temperature for 30 min. The *T*_2_ relaxation time was measured by the Carr–Purcell–Meiboom–Gill (CPMG) sequence with 8 scans, 5000 echoes, and 4000 ms between scans. The raw data were analyzed by a multi-exponential fitting model with MultiExp Inv Analysis software, V4.0 (Niumag Analytical Instrument Corporation, Suzhou, China), to obtain the transverse relaxation time (*T*_21_, *T*_22_, and *T*_23_) and peak area proportion of the corresponding water populations (*P*_21_, *P*_22_, and *P*_23_).

### 2.10. Determination of Chemical Interaction

Chemical interactions were determined by referring to the measurements adopted by Zhao et al. and Khoder et al., with appropriate adjustments [26,29]. The cooked samples were dispersed in different reagents: 0.05 M of NaCl (RA), 0.6 M of NaCl (RB), 0.6 M of NaCl + 1.5 M of urea (RC), 0.6 M of NaCl + 8 M of urea (RD), and 0.6 M of NaCl + 8 M of urea + 0.5 M of β-mercaptoethanol (RE), and then the soluble protein concentrations (g/L) were determined. All reagents were prepared with 0.05 M of a phosphate buffer (with a pH of 7.0). Briefly, approximately 2 g of samples were homogenized in 10 mL of the solution above for 2 min, followed by stirring at 4 °C for 1 h and centrifuging twice at 12,000× *g* and 4 °C for 15 min each. The differences in protein concentration in different reagents represent the contents of various chemical forces, and differences between RB and RA, between RC and RB, between RD and RC, and between RE and RD represent the content of ionic bonds, hydrogen bonds, hydrophobic interactions, and disulfide bonds in the protein solution, respectively.

### 2.11. Fourier Transform Infrared Spectroscopy (FT-IR)

The secondary structures of cooked 3D samples were analyzed, referring to the method described by Zhang et al., with slight modifications [27]. The cooked samples were freeze-dried at −50 °C for 48 h. Afterward, the samples were mixed with KBr (1:100, *w*/*w*) and ground uniformly, then compressed into a translucent sheet. The spectra data were collected using an FT-IR spectrometer (Vertex 70V, Bruker Optics, Karlsruhe, Germany), scanning 32 times in the wave-number range of 4000–400 cm^−1^. Finally, according to the spectra data, the contents of the β-sheet, random coil, α-helix, and β-turn were estimated, respectively, with OMNIC software, V8.2 (Thermo Fisher Scientific, Madison, WI, USA), and PeakFit software, V4.4 (SeaSolve Software Inc., Framingham, MA, USA).

### 2.12. Scanning Electron Microscopy (SEM)

According to the method of Zhang et al., with appropriate adjustment, the microstructure of 3D-printed samples was observed using an SEM (SU8000, Hitachi Co., Tokyo, Japan) [27]. Samples were sliced into about 2-mm sheets and freeze-dried at −50 °C. Afterward, the freeze-dried samples were fixed on the sample table, sprayed with gold, and observed at an accelerating voltage of 5 kV. Finally, photographs at a magnification of 5.0 k were collected.

### 2.13. Statistical Analysis

All experimental data were expressed as the mean ± standard deviation (SD). Differences were tested for significance by one-way analysis of variance (ANOVA) and performed by using IBM SPSS Statistics 26 for Windows (SPSS Inc., Chicago, IL, USA). Trends were considered significant at *p* < 0.05. OriginPro 2021 (OriginLab, Northampton, MA, USA) was used for further analysis and plotting of data subsequently.

## 3. Results and Discussion

### 3.1. Rheological Properties

**Figure 1:**
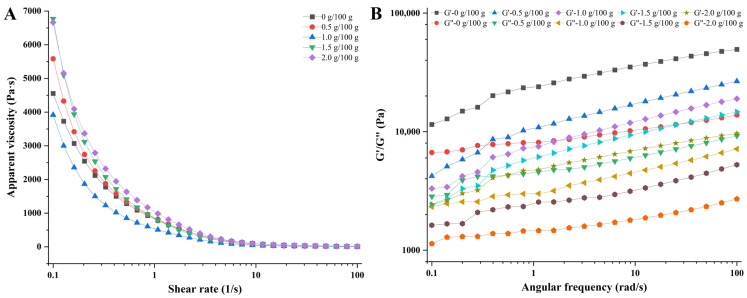
Rheological properties of surimi gels containing 0, 0.5, 1.0, 1.5, and 2.0 g/100 g of CaCl_2_. (**A**) Apparent viscosity; (**B**) storage modulus (G′) and loss modulus (G″).

The printability and shape retention ability of 3D printing inks are closely related to their rheological properties [30]. The flow behavior and loss modulus of the material affect its extrusion properties, while the storage modulus affects its ability to retain the shape of the printed product [31]. As shown in Figure 1A, the apparent viscosity of surimi gels with different concentrations of CaCl_2_ added decreased with an increase in the shear rate, suggesting that the surimi gels were a non-Newtonian fluid with shear-thinning behavior [32]. The similar flow behaviors indicated that the surimi gels could be smoothly squeezed out from the printer nozzle [33]. Moreover, the apparent viscosity of the inks increased overall with the addition of CaCl_2_, while Wang et al., who found that adding NaCl reduced the viscosity of surimi gels in their study, explained that NaCl could dissolve the myofibrillar proteins and induce unfolding and re-arrangement of the protein structures, thus reducing the viscosity of surimi gels [32]. The addition of CaCl_2_ could also promote the unfolding of the protein structures differently; the proteins will form an inter-molecular cross-linking structure in the presence of TGase, thereby increasing the viscosity of the surimi gel system [21]. On the other hand, the effect of ionic bonds in the surimi gel system became more prominent (possibly forming calcium bridges) with the increasing concentration of CaCl_2_, prompting further aggregation of the protein molecules and resulting in increasing the viscosity of the gel. The appropriate elevation of the apparent viscosity is conducive to improving the consistency of the 3D printing product molding [15]. Interestingly, the viscosity of the gel decreased at 1.0 g/100 g of CaCl_2_-addition, which was possibly attributed to the formation of a uniform network structure among the protein molecules, making the water molecules more evenly distributed in the pores and thus reducing the viscosity of the gel system [34].

The printability and the self-support property of materials are closely related to their rheological properties. The storage modulus (G′) reflects the solid-like behavior of the material, which is related to the self-support property of the material, while the loss modulus (G″) reflects the liquid-like behavior of the material and is related to the extrusion property of the material [34,35]. As shown in Figure 1B, the G′ and G″ of the gels gradually decreased with the concentration of CaCl_2_ increasing, indicating that the mechanical properties of the surimi gels had changed (the rigidity decreased) [36]. In addition, within the range of linear viscoelasticity, the storage modulus of the surimi gels with different treatments was greater than the loss modulus, indicating that the gels have elastic gel properties [37]. In other words, the result of G′ > G″ suggests that the inks could be extruded smoothly from the printing nozzle and perform good self-support and stability after the extrusion [38].

### 3.2. 3D Printing Performance

**Figure 2:**
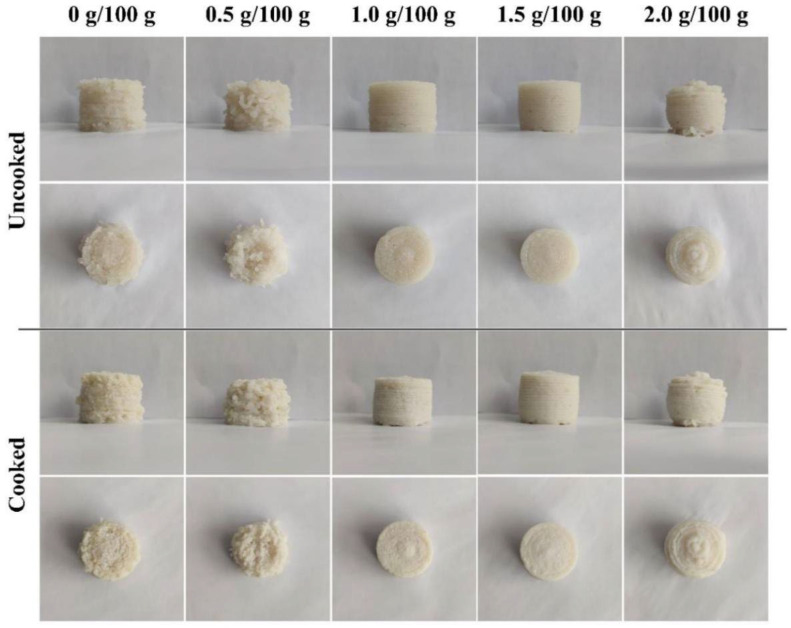
3D printing performance of surimi gels incorporated with CaCl_2_ at different concentrations.

Digital photographs of the 3D-printed products are shown in Figure 2. When the amount of CaCl_2_ was less than 1.0 g/100 g, the extrusion lines of the surimi gels were discontinuous, and the surface of the printing products was rough, which might be attributed to the formation of the rigidity and fragility structures caused by the addition of CaCl_2_ [39]. When the content of the CaCl_2_ increased to 1.0 g/100 g and 1.5 g/100 g, the extrusion lines of the inks became smoother and more continuous, the texture of the printed products became clearer, and the model reduction degree became higher gradually. However, when the addition amount of CaCl_2_ was increased to 2.0 g/100 g, the gel became difficult to squeeze out from the nozzle, and the extrusion speed no longer matched the printing speed, and then the dragging phenomenon happened, resulting in an imprecise layer deposition and printing shape deformation. The viscosity and fluidity of the surimi gel affect its 3D printing performance. When the viscosity of the materials is too low, the extrusion lines will be discontinuous, and the ability of layer deposition and coherence will be insufficient, resulting in a rough surface and poor printing accuracy. On the contrary, when the viscosity is too high, the fluidity of the materials will deteriorate, the extrusion of the materials becomes more difficult, and the extrusion rate cannot match the set parameters, resulting in uneven extrusion and poor printing performance.

### 3.3. Printing Precision and Shrinkage Rate

**Figure 3:**
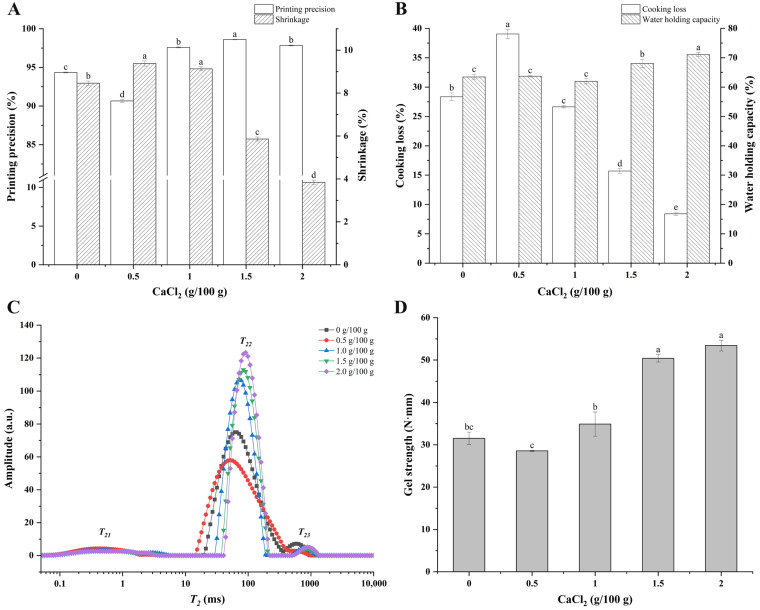
Printing precision and shrinkage (**A**), cooking loss and water-holding capacity (**B**), distribution of *T*_2_ relaxation time (**C**), and gel strength (**D**) of 3D-printed surimi gels incorporated with CaCl_2_ at different concentrations. Different lowercase letters above the error bar in (**A**,**B**,**D**) indicate significant differences (*p* < 0.05) among various printed surimi gels incorporated with CaCl_2_ at different concentrations.

Printing precision is a quantitative index of printing performance, which can effectively reflect the degree of ink restoring the 3D model after 3D printing and is related to the rheological characteristics of the materials [40]. As shown in Figure 3A, the printing precision of surimi gel with 0.5 g/100 g of CaCl_2_ added decreased from 94.34% to 90.66% (*p* < 0.05) compared with the control group, which was possibly related to changes in the mechanical properties and viscosity. With the content of the CaCl_2_ increasing, the printing precision was significantly enhanced (*p* < 0.05), and the printing precision of surimi gel with 1.5 g/100 g of CaCl_2_ added reached the highest value of 98.61%. After the cooking, the geometry of the printed surimi gel samples shrank to varying degrees due to the thermal denaturation and contraction of the protein [41]. As shown in Figure 3A, the size of the samples shrank differently after the heating treatment, and the shrinkage rate was negatively correlated with the concentration of the CaCl_2_. The shrinkage rate of surimi gel with 2.0 g/100 g of CaCl_2_ added reached the minimum value of 3.84%, suggesting that the addition of CaCl_2_ made the products have a stronger shape stability and effectively reduced the adverse effects of the heat treatment. This might be attributed to the intra- and inter-molecular interactions and covalent cross-linking formed after the dissolution of the myofibrillins [37].

### 3.4. Cooking Loss and WHC

The cooking loss and WHC of the 3D-printed samples are shown in Figure 3B. The index of cooking loss is often used to evaluate the loss of water, protein, and other nutrients during the ripening process of meat products [15]. Compared with the control group, the cooking loss of the sample with the addition of 0.5 g/100 g of CaCl_2_ significantly increased from 28.36% to 39.05% (*p* < 0.05), which was probably attributed to the myofibrillins not fully unfolding, leaving it weakly bound to the water molecules, or the structure of the proteins contracting and accumulating to expel the water under the action of heating [37]. In addition, the rough surface and porous interior structure could have caused the loss of more water, protein, and other substances. When the content of the CaCl_2_ exceeded 0.5 g/100 g, the cooking loss of the gels was significantly reduced (*p* < 0.05), and the cooking loss of the gels with 1.5 g/100 g and 2.0 g/100 g of CaCl_2_ added declined to 15.70% and 8.41%, respectively, which might have benefited from the smoother surface and more dense internal structure of the printed products.

WHC is often used to evaluate the quality and structural strength of meat products [15]. As shown in Figure 3B, there is no significant difference in the WHC of the samples (*p* > 0.05) with 0, 0.5, and 1.0 g/100 g of CaCl_2_ added. The WHC of the samples increased significantly (*p* < 0.05) once the concentration of CaCl_2_ increased to 1.5 g/100 g, and the WHC of the gels with an added 1.5 and 2.0 g/100 g of CaCl_2_ reached 68.07% and 71.09%, respectively, which could be explained by the increase of hydrogen bond forces in the gels. In other words, the structure of the proteins was expanded with the addition of the CaCl_2_, which promoted the interaction between the water molecules and protein molecules, while the formation of the network structure also made the water molecules more firmly restricted.

### 3.5. T_2_ Relaxation Time of Surimi Gels

Migration and absorption information of the water molecules in the cooked samples were analyzed with LF-NMR, and the results are shown in Figure 3C and Table 1. There were three water populations in each sample centered at 0–10 ms (*T*_21_), 30–200 ms (*T*_22_), and 200–1000 ms (*T*_23_), respectively [21]. Generally, *T*_21_ represents the water that combined tightly within the protein molecule and participated in the structure of the protein, *T*_22_ mainly corresponds to the water entrapped within the gel networks and the water in the myofibrils, which is also known as immobilized water [42], and *T*_23_ corresponds to the free water mainly retained in large pores within the gel network structures, which is easy to lose in the process of heating [27,41]. As shown in Figure 3C, the immobilized water was the main component of the water in the surimi gel, and the *T*_2_ relaxation time distribution of the samples was increasingly concentrated, and the signal value gradually strengthened, suggesting that water migration was hindered and resulting in the increased mechanical strength of the surimi gel [39]. According to Table 1, the *T*_21_ relaxation time of the sample did not change significantly (*p* > 0.05) with the concentration of the CaCl_2_ increasing, while the *T*_22_ and *T*_23_ relaxation times were postponed significantly (*p* < 0.05). This variation in the *T*_2_ relaxation time was related to the aggregation of protein and formation of gels. However, a different result was observed for the sample with 0.5 g/100 g of CaCl_2_, which could have been the result of the loss of more water from its loose and porous internal structure after the heating treatment.

The peak proportions of the *T*_2_ relaxation time of the samples are shown in Table 1. Compared with the control group, the significant increase of *P*_21_ in the gel with 0.5 g/100 g of CaCl_2_ added might be attributed to the protein molecules binding more water molecules during the rapid aggregation. With the concentration of CaCl_2_ gradually increased to 2.0 g/100 g, the *P*_21_ and *P*_23_ of the surimi gel decreased, while *P*_22_ increased significantly (*p* < 0.05), which indicated that more bound water or free water transformed into immobilized water with the addition of CaCl_2_, suggesting that more hydrogen bonds might have been formed in the gel system. The phenomenon of water migration such as this would have a positive effect on the WHC of surimi gels [15].

### 3.6. Gel Strength

Gel strength is an important indicator in assessing the quality of fish products. The gel strength of the cooked samples is shown in Figure 3D. Compared to the control group, the gel strength of the samples decreased first and then increased with the increasing addition of CaCl_2_. The gel strength of the printed product with an added 2.0 g/100 g of CaCl_2_ reached the maximum value of 53.41 N·mm, which was 1.69 times that of the control group (*p* < 0.05). NaCl promoted the protein structure unfolding and exposed the functional groups hidden inside the protein, thus enhancing the inter-molecular interaction force and enabling the control sample with a relatively high gel strength [43]. In the group with an added 0.5 g/100 g of CaCl_2_, the protein might have aggregated rapidly, forming a rough, porous structure and resulting in lower gel strength. As the concentration of CaCl_2_ increased unceasingly, the formation of the inter-molecular calcium bridge between the Ca^2+^ and the COO^−^ on the protein side-chain promoted the formation of a three-dimensional network structure with a higher density and lower porosity, thus enhancing its gel strength [44].

### 3.7. Color and TPA

Color is an important indicator of determining the sensory quality of products, and it affects consumers’ desire to choose. For fish products, consumers prefer products with a higher whiteness value [15]. As shown in Table 2, the a* values and b* values of the samples with the addition of CaCl_2_ decreased significantly (*p* < 0.05), while the brightness values and whiteness values increased significantly (*p* < 0.05). After the concentration of CaCl_2_ increased to 1.0 g/100 g, the brightness and whiteness increased slowly, indicating that the effect of CaCl_2_ on the color of surimi gels had reached an equilibrium point, which could be related to the change in the microstructure of surimi gels. Feng et al. found that the whiteness of gel samples was also improved after adding CaCl_2_ and indicated that the products were more likely to be favored by consumers [13].

Similarly, textual characteristics also reflect the consumers’ acceptance of the products [16]. As shown in Table 3, the hardness, springiness, and chewiness values of the samples first decreased and then increased with the addition of CaCl_2_. A relatively low concentration of CaCl_2_ increased the rigidity of the surimi gels, while the sample with 1.0 g/100 g of CaCl_2_ added had minimum textual values, which could be attributed to the formation of a more tender texture due to more water molecules trapped in the pores of the gel network. As the content of CaCl_2_ increased to 1.5 g/100 g, the hardness and chewiness values of the sample reached 48.18 N and 31.00 N, respectively, which were 2.40 times and 4.42 times that of the control group (*p* < 0.05), respectively. In addition, the texture characteristics changed lightly with the continuous addition of CaCl_2_ (*p* > 0.05). Moreover, the springiness of the product with 1.5 g/100 g of CaCl_2_ incorporated was significantly increased to 0.89 (*p* < 0.05), indicating that the product had a stronger elastic texture, which may be more interesting to consumers.

### 3.8. Chemical Interactions

The effects of the CaCl_2_ content on the non-covalent bonds and disulfide bonds in the surimi gel are presented in Table 4. As we expected, the ionic bonds’ content in the surimi gels increased significantly (*p* < 0.05) with the increasing amount of CaCl_2_. The hydrogen bonds’ content also increased with the addition of CaCl_2_, which could be explained by more water molecules being entrapped in the better-organized gel network. The increased ionic bonds’ content contributed to improvement in the flow behavior of the gel, while the increased hydrogen bonds’ content enhanced the gel strength [45]. In addition, as shown in Table 4, hydrophobic interactions and disulfide bonds were the main non-covalent forces in the surimi gels; the contents all showed a downward trend with the increasing of CaCl_2_ concentrations, except for a significant increase (*p* < 0.05) in the hydrophobic interactions of the surimi gel with 0.5 g/100 g of CaCl_2_ added. The increase in the hydrophobic interactions’ content might be related to the rapid aggregation of the proteins, while the decrease in the hydrophobic interactions’ and disulfide bonds’ contents might be related to the formation of inter-molecular calcium bridges and non-disulfide covalent bonds.

### 3.9. Chemical Structure of Surimi Gel Proteins

**Figure 4:**
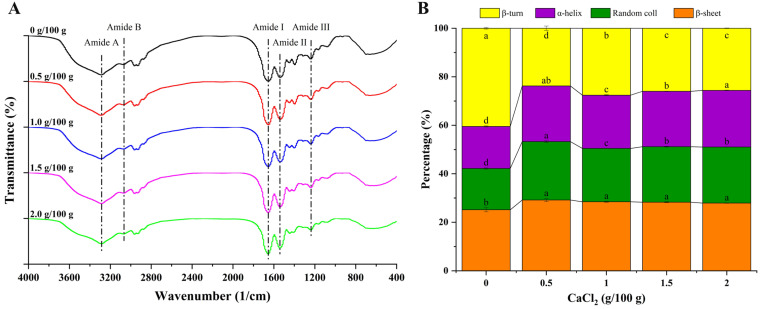
Fourier transform infrared (FT-IR) spectra (**A**) and calculated protein secondary structures (**B**) of 3D-printed surimi gels incorporated with CaCl_2_ at different concentrations. Different letters in (**B**) indicate significant differences (*p* < 0.05).

The infrared spectrum and protein secondary structure of the cooked surimi gels are shown in Figure 4A,B, respectively. The bands at 1700–1600 cm^−1^, 1600–1500 cm^−1^, and 1330–1220 cm^−1^ are amide I, II, and III bands of proteins, respectively, while the bands at 3600–3200 cm^−1^ and 3100–3000 cm^−1^ are amide A and B, respectively [27,46]. As shown in the spectrum, there was no significant difference in the infrared characteristic absorption peaks of the different formulated gel samples, indicating that the surimi gel did not produce new functional groups after the addition of calcium chloride. In the spectrum, the wide and strong absorption band at 3600–3200 cm^−1^ corresponds to the stretching vibration of O-H bonds, indicating the existence of hydrogen bonding in the surimi gel system [27,29]. The control group had a characteristic absorption peak at 3289 cm^−1^, while the characteristic absorption peak of the gel samples supplemented with 0.5, 1.0, 1.5, and 2.0 g/100 g of CaCl_2_ was at 3290, 3288, 3287, and 3286 cm^−1^, respectively. Compared with the control group, the characteristic absorption peaks shifted slightly to the shorter wave-number for the gel samples with 1.0, 1.5, and 2.0 g/100 g of CaCl_2_ added, indicating that stronger hydrogen bonding formed in the gel system, which might be one reason for the increase in the viscosity of the gel with the rising of the CaCl_2_ concentration [39].

The area proportions of the protein secondary structure were estimated by Gaussian deconvolution and the second derivative fitting of the amide I band (1700–1600 cm^−1^), and the results are shown in Figure 4B. The corresponding relationship between each subpeak and the protein secondary structure is as follows: 1695–1660 cm^−1^ corresponds to the β-turn, 1660–1650 cm^−1^ corresponds to the α-helix, 1650–1640 cm^−1^ corresponds to the random coil, and 1640–1600 cm^−1^ corresponds to the β-sheet [47]. The β-turn content gradually decreased from 40.47% to 25.98% with the additive amount of CaCl_2_ up to 1.5 g/100 g (*p* < 0.05), while the content of the α-helix (from 17.36% to 22.84%), β-sheet (from 25.16% to 28.28%), and random coil (from 17.01% to 22.90%) increased significantly (*p* < 0.05). The inter-molecular ordering arrangement of proteins is maintained by the α-helix through inter-molecular hydrogen bonds, while the intra-molecular ordering arrangement of proteins is maintained by the β-sheet through intra-molecular hydrogen bonds [48]. The decrease in the β-turn content and the increase in the α-helix and β-sheet content suggested that the secondary structures of the proteins were re-arranged after unfolding and formed a more ordered structure [13]. Notably, in the group with the addition of 0.5 g/100 g of CaCl_2_, the α-helix, β-sheet, and random coil content (22.95%, 29.16%, and 24.10%, respectively) were significantly higher than in the control and other experimental groups (*p* < 0.05), while the β-turn content (23.79%) was significantly lower than in the other groups (*p* < 0.05). These results indicated that the proteins accumulated rapidly before totally unfolding and formed a disordered structure, which could explain the poor printing performance, high cooking loss, and weak gel strength of surimi gel with 0.5 g/100 g of CaCl_2_ added.

### 3.10. Microstructure of Surimi Gel

**Figure 5:**
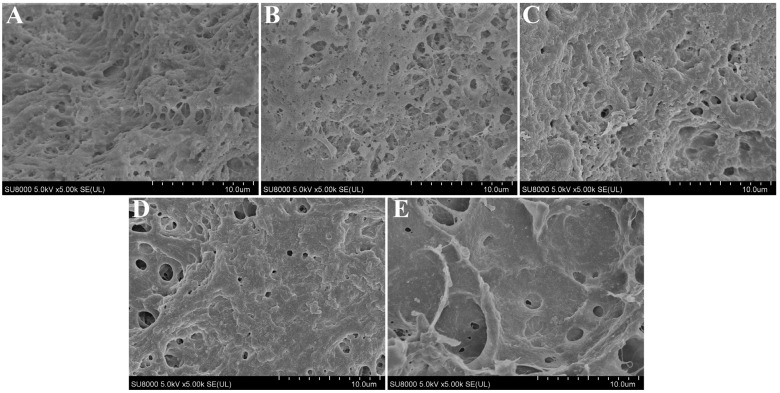
Microstructure micrographs (5.0 k) of 3D-printed surimi gels incorporated with CaCl_2_ at different concentrations. (**A**–**E**): the concentrations of incorporated CaCl_2_ were 0, 0.5, 1.0, 1.5, and 2.0 g/100 g, respectively.

The microstructure of the cooked surimi gels is illustrated in Figure 5. As per the results predicted by the 3D printing performance (Figure 2), the microstructures of surimi gel with the added 0 and 0.5 g/100 g of CaCl_2_ were loose, disordered, and had more pores (Figure 5A,B), which might be the result of the rapid aggregation of the protein molecules before fully unfolding. When 1.0 g/100 g of CaCl_2_ was added, a relatively ordered network structure with many small pores was formed inside the gel product (Figure 5C), which was conducive to entrapping more water molecules into the pores after the heating and cooling [15,44], thus resulting in a higher hydrogen bonds content. However, this structure was more likely to allow water to be lost under the action of a strong external force (the centrifugal force), resulting in water loss, which could be the reason for its lower water-holding capacity. With the concentration of CaCl_2_ continuing to increase (>1.0 g/100 g), the protein molecules continued to unfold and re-arrange, and then a more orderly cross-linking structure formed between the protein molecules, with the effects of the calcium bridges and non-disulfide covalent bonds. Finally, a highly cross-linked and more compact and uniform three-dimensional network structure with fewer pores was formed (Figure 5D,E), which could explain the higher apparent viscosity, gel strength, and hardness of the gels [39].

## 4. Conclusions

This study found that adding an appropriate amount of CaCl_2_ could not only effectively compensate for the defective gel performance of low-salt surimi gel but also improve the 3D printing quality of the gel and enhance the mechanical strength and stability of the printed product after heating. Furthermore, the addition of CaCl_2_ increased the whiteness value of the surimi products, resulting in improving their sensory quality. The results of the rheology and 3D printing showed that adding 1.5 g/100 g of CaCl_2_ could improve the apparent viscosity and reduce the storage modulus and loss modulus, which led to the improvement of the 3D printing performance. The results of the chemical interactions, chemical structure, water distribution, and microstructure of the 3D-printed products showed that adding 1.5 g/100 g of CaCl_2_ could enhance the WHC and mechanical strength (the gel strength, hardness, springiness, etc.) and depress the cooking loss by unfolding the protein structure and forming an orderly and uniform three-dimensional network structure, resulting in better sensory properties. In conclusion, CaCl_2_ can be used as a salt substitute in the production of surimi products, which could provide a theoretical basis for the development of healthy and nutritious 3D foods.

## Figures and Tables

**Table 1:** LF-NMR spin–spin relaxation time (*T*_2_) and peak area proportion of the three populations of the 3D-printed surimi gels incorporated with CaCl_2_ at different concentrations.

CaCl_2_ (g/100 g)	*T*_21_ (ms)	*T*_22_ (ms)	*T*_23_ (ms)	*P*_21_ (%)	*P*_22_ (%)	*P*_23_ (%)
0	2.05 ± 0.04 ^a^	38.45 ± 1.05 ^d^	361.12 ± 0.00 ^c^	6.58 ± 0.10 ^b^	89.76 ± 0.11 ^d^	3.66 ± 0.06 ^a^
0.5	1.78 ± 0.19 ^a^	31.81 ± 0.00 ^e^	307.88 ± 14.39 ^d^	7.45 ± 0.08 ^a^	91.17 ± 0.11 ^c^	1.38 ± 0.12 ^c^
1.0	1.96 ± 0.01 ^a^	43.98 ± 0.00 ^c^	500.54 ± 23.39 ^b^	6.13 ± 0.06 ^c^	91.85 ± 0.31 ^b^	2.02 ± 0.25 ^b^
1.5	2.00 ± 0.13 ^a^	51.71 ± 0.00 ^b^	527.54 ± 14.05 ^ab^	5.44 ± 0.25 ^d^	92.81 ± 0.30 ^a^	1.75 ± 0.08 ^bc^
2.0	1.80 ± 0.18 ^a^	56.07 ± 0.00 ^a^	556.82 ± 15.23 ^a^	5.38 ± 0.09 ^d^	92.44 ± 0.06 ^ab^	2.18 ± 0.08 ^b^

All data are shown as means ± SD (*n* = 3). For the same column, different letters indicate significant differences (*p* < 0.05).

**Table 2:** The color of 3D-printed surimi gels incorporated with CaCl_2_ at different concentrations.

CaCl_2_ (g/100 g)	L*	a*	b*	Whiteness
0	76.86 ± 0.10 ^c^	−0.52 ± 0.04 ^a^	11.99 ± 0.14 ^a^	73.93 ± 0.14 ^d^
0.5	78.44 ± 0.32 ^b^	−0.67 ± 0.05 ^b^	11.14 ± 0.12 ^b^	75.72 ± 0.27 ^c^
1.0	82.28 ± 0.20 ^a^	−0.92 ± 0.02 ^c^	10.61 ± 0.08 ^c^	79.32 ± 0.19 ^b^
1.5	82.18 ± 0.21 ^a^	−1.35 ± 0.02 ^d^	9.82 ± 0.13 ^d^	79.61 ± 0.24 ^b^
2.0	82.56 ± 0.23 ^a^	−2.27 ± 0.04 ^e^	8.72 ± 0.27 ^e^	80.36 ± 0.15 ^a^

All data are shown as means ± SD (*n* = 3). For the same column, different letters indicate significant differences (*p* < 0.05).

**Table 3:** Texture characteristics of 3D-printed surimi gels incorporated with CaCl_2_ at different concentrations.

CaCl_2_ (g/100 g)	Hardness (N)	Springiness	Cohesiveness	Chewiness (N)	Resilience
0	20.05 ± 0.15 ^b^	0.85 ± 0.02 ^b^	0.41 ± 0.01 ^b^	7.02 ± 0.23 ^b^	0.15 ± 0.00 ^c^
0.5	16.59 ± 0.76 ^c^	0.84 ± 0.01 ^b^	0.41 ± 0.02 ^b^	5.75 ± 0.41 ^c^	0.18 ± 0.01 ^b^
1.0	16.47 ± 0.78 ^c^	0.74 ± 0.02 ^c^	0.32 ± 0.01 ^c^	3.85 ± 0.32 ^d^	0.11 ± 0.00 ^d^
1.5	48.18 ± 0.26 ^a^	0.89 ± 0.01 ^ab^	0.72 ± 0.00 ^a^	31.00 ± 0.41 ^a^	0.36 ± 0.00 ^a^
2.0	46.89 ± 0.60 ^a^	0.90 ± 0.00 ^a^	0.74 ± 0.00 ^a^	31.04 ± 0.51 ^a^	0.37 ± 0.00 ^a^

All data are shown as means ± SD (*n* = 3). For the same column, different letters indicate significant differences (*p* < 0.05).

**Table 4:** The changes of chemical interactions of 3D-printed surimi gels incorporated with CaCl_2_ at different concentrations.

CaCl_2_ (g/100 g)	Ionic Bonds (g/L)	Hydrogen Bonds (g/L)	Hydrophobic Interactions (g/L)	Disulfide Bonds (g/L)
0	0.33 ± 0.01 ^c^	0.45 ± 0.02 ^d^	5.18 ± 0.16 ^b^	5.76 ± 0.06 ^a^
0.5	0.38 ± 0.02 ^bc^	0.38 ± 0.02 ^d^	7.16 ± 0.07 ^a^	5.46 ± 0.08 ^a^
1.0	0.45 ± 0.03 ^b^	0.98 ± 0.02 ^a^	5.21 ± 0.04 ^b^	5.62 ± 0.17 ^a^
1.5	0.60 ± 0.03 ^a^	0.61 ± 0.04 ^c^	4.11 ± 0.10 ^c^	4.22 ± 0.15 ^b^
2.0	0.40 ± 0.03 ^bc^	0.85 ± 0.04 ^b^	3.46 ± 0.08 ^d^	3.22 ± 0.07 ^c^

All data are shown as means ± SD (*n* = 3). For the same column, different letters indicate significant differences (*p* < 0.05).

## Data Availability

Data is contained within the article.
